# Livestock guinea pigs: a scoping review from a One Health perspective

**DOI:** 10.3389/fvets.2026.1830046

**Published:** 2026-08-03

**Authors:** Mauricio Salas-Rueda, Angel Sebastián Rodríguez-Pazmiño, Melissa Joseth Carvajal-Capa, José Julián Zúñiga-Velarde, Solon Alberto Orlando, Miguel Angel García-Bereguiain

**Affiliations:** 1 Globalgen, Universidad Politécnica Salesiana, Cuenca, Ecuador; 2 One Health Research Group, Universidad de Las Américas, Quito, Ecuador; 3 Universidad Católica Santiago de Guayaquil, Guayaquil, Ecuador; 4 Universidad Ecotec, Samborondón, Ecuador; 5 Instituto Nacional de Salud Pública e Investigación, Guayaquil, Ecuador

**Keywords:** Andean Region, Ecuador, guinea pig farming, leptospirosis, One Health, Peru, toxoplasmosis

## Abstract

The guinea pig (*Cavia porcellus*) has been raised in the Andean Region of South America since ancient times, valued not only for its nutritional contributions but also for its cultural and religious significance. The guinea pig remains a significant source of food and incomes for rural communities in the Andean Region of South America, including Colombia, Ecuador, Peru and Bolivia. However, while many publications are available about infectious diseases associated to guinea pigs either as laboratory animal model or pets, this knowledge is scarce for guinea pigs raised as livestock. Guinea pig production remains mainly traditional without specific animal health guidelines or zoonotic surveillance programs compared to cattle, pigs or poultry. The aim of this scoping review is to analyze the available literature related to the potential role of livestock guinea pigs as reservoir for zoonotic transmission. Several reports have already shown the carriage of respiratory and enteric bacterial and parasites, and also other pathogens responsible for zoonotic diseases like toxoplasmosis, leptospirosis or bartonelosis. Guinea pig farming industry have to improve an integrative animal health and production strategy from a One Health perspective to reduce the occupational risk and guarantee food security.

## Introduction

The guinea pig (*Cavia porcellus*) has been raised in the Andean Region of South America since ancient times, valued not only for its nutritional contributions but also for its cultural and religious significance. During the Inca Empire, the guinea pig was considered sacred and played a central role in rituals and religious ceremonies, and also a crucial source of protein (1). Beyond its spiritual and dietary roles, the guinea pig has held an important place in traditional medicine. Even today, some Andean communities use guinea pigs in healing rituals, reflecting the enduring influence of Inca traditions (2).

In a different context, guinea pigs are also widely kept as recreational animals (pets) in various parts of the world (3, 4). This distinct purpose differs significantly from the South American realities, yet it represents another productive activity centered around guinea pigs (3, 4).

In addition, the guinea pig has become one of the most prominent mammals in scientific research worldwide. It is among the most extensively studied animal species, contributing notably to advancements in science and medicine (5, 6). The guinea pig has been integral to multidisciplinary studies exploring the pathogenic mechanisms of human and zoonotic diseases, the development of vaccines, diagnostic tests, pharmaceuticals, and more. Its unique anatomical and physiological characteristics make it an exceptional animal model for experimentation (5, 6).

Native to South America and primarily found in the Andean Regions of Peru, Bolivia, Ecuador, and Colombia, the guinea pig has served as food source since pre-Inca times. Its domestication is believed to have occurred between 3,000 and 5,000 years ago (7). Despite the introduction of other livestock species following the arrival of the Spaniards, guinea pigs remained a vital resource for indigenous communities, particularly in the high Andean Regions. Their importance endured due to their rapid reproductive cycle and remarkable ability to thrive in challenging geographical and climatic conditions (7). Today, Peru stands as the leading producer and consumer of guinea pigs in the region, although production in Colombia, Peru and Boliva are also significant. For instance, only in Ecuador at least 700,000 families are involved in guinea pig farming with an estimated annual production of 47 million according to data from the *Instituto Nacional de Investigaciones Agropecuarias* (8). Despite its importance to the rural local economy, its breeding in small scale farms is still performed traditionally without the use of technology and proper conditions required not only to reach high levels of meat production but also to guarantee food safety and quality (8, 11). Nevertheless, beyond being a protein-rich food source, guinea pigs now serve as an economic asset for many rural families in Andean countries, driving agro-industrial development and supporting local trade.

In recent decades, guinea pig production in Latin America has undergone significant transformation, shifting from traditional practices to more technological systems at large scale farms. This modernization aims to meet the growing demand for guinea pig meat, particularly in the Andean countries, by maximizing productivity and ensuring the sustainability of the activity (12). Inspired by the advancements in poultry and pig production since the 1970s, proven techniques from these industries have been adapted for guinea pig farming. Improvements in nutrition, genetics, and management have led to enhanced growth rates, greater reproductive efficiency, and reduced mortality on guinea pig farms The introduction of specific nutritional supplements and balanced diets has further optimized feed conversion rates, significantly boosting overall productivity (12). The selection and genetic improvement of guinea pig lines with desirable traits, such as larger size, rapid growth, and disease resistance, have played a key role in advancing production (7). In addition to genetic advancements, technification has focused on improving production environments by incorporating climate control in sheds, automated feeding and watering systems and improving hygiene. These measures have significantly enhanced the health and welfare of the animals (12).

The meat of *Cavia porcellus* is widely valued for its exceptional nutritional properties, offering a rich source of protein with low-fat content. Guinea pig meat contains approximately 18% protein, making it a valuable dietary option in regions where malnutrition is a pressing concern (13). Moreover, its high-quality essential amino acid profile compares favorably to other animal protein sources such as beef and chicken, further highlighting its nutritional significance (14).

Proper health management is essential for maintaining the quality of guinea pig meat. Guinea pigs are susceptible to various diseases, which can not only reduce productivity but also compromise the food safety of the final product. In this sense, implementing rigorous management practices, including strict hygiene measures and balanced feeding are mandatory (14). These practices are vital to preserving the nutritional quality of the meat and maximizing its benefits for the communities that rely on guinea pigs as a critical source of protein. Implementing proper hygiene measures is a major challenge in guinea pig management due to their high susceptibility to bacterial and parasitic diseases (14). The lack of training in hygienic management practices and early disease detection remains a critical issue for low and medium scale production farms of less than a thousand animals.

Despite those challenges, the global demand for guinea pig meat is growing, driven by increasing interest in sustainable and healthy meat alternatives. In Peru, annual guinea pig consumption exceeds 65 million animals, while in Ecuador, it reaches 47 million, underscoring the cultural and dietary significance of this meat in the Andean Region. Internationally, the U.S. market has shown remarkable growth, fueled by rising consumer interest and the influence of Andean communities. Market projections suggest that demand for guinea pig meat could increase further in the coming years, thanks to its favorable nutritional profile and targeted marketing efforts positioning it as a premium option in restaurants and specialty supermarkets (15). The international market for guinea pig meat has experienced steady growth, particularly in regions outside South America. In the United States, “Latino supermarkets” have whole and frozen guinea pigs available, priced between $25 and $35 per unit, reflecting both the growing demand and the premium quality associated with this product (15). In this sense, to develop proper hygiene guidelines for guinea pigs production and management are needed more than ever to prevent public health issues beyond the Andean countries.

So far, the guinea pig remains a significant source of food and incomes for rural communities in the Andean Region of South America. Its breeding provides rural families with a reliable source of income, while its meat is highly valued for its exceptional nutritional content. In many developing countries facing food security challenges, there is an urgent need to identify sustainable protein sources. Guinea pigs have been evaluated as a viable and promising alternative due to their ability to efficiently convert low-quality food resources into high-value protein, their rapid reproduction rate, and their natural resistance to disease (16). Due to the pressing need for high-quality, low-cost protein, several countries in Africa have embraced guinea pig production in recent years. Nations such as Cameroon, the Democratic Republic of Congo, and Ghana have adapted production systems tailored to their local realities. These systems are modeled after South American management practices, focusing on promoting human development, ensuring food sovereignty, and generating economic income for families, particularly those from vulnerable socio-economic backgrounds (17).

However, while many publications are available about infectious diseases associated to guinea pigs either as laboratory animal model or pets, this knowledge is scarce for guinea pigs raised as livestock. Guinea pig production remains mainly traditional without specific animal health guidelines or zoonotic surveillance programs compared to cattle, pigs or poultry. Additionally, as guinea pig production is mainly focused in low and middle income countries (LMICs) of the Andean Region (Colombia, Ecuador, Peru and Bolivia) and some African countries, research is also limited due to budget constraints. From a food security and public health perspective, important challenges arise when livestock guinea pigs are analyzed through the lens of a One Health approach as potential reservoirs for zoonotic transmission. The holistic framework of One Health systematically examines the complex interconnections between humans, animals, and the environment, offering effective and integrative strategies to address food safety and public health concerns (18). In this sense, there is a large literature about the use of guinea pigs as animal model for infectious diseases. There are also many scientific reports about zoonotic spillovers from guinea pigs raised as pets. However, as stated above, the research about zoonotic diseases affecting livestock guinea pigs is more limited.

Under this scenario, the aim of this scoping review was to study the available literature about livestock guinea pig animal health, focusing on its role as a reservoir for zoonotic transmission and potential pathways to improve livestock guinea pig health and production from a One Health perspective.

## Bibliographic search strategy

This scoping review was conducted using a systematic search strategy across the databases SCOPUS, Web of Science, and SciELO for articles written in English and Spanish, restricted to the period 2000–2025; those databases were selected for their extensive coverage of international and regional scientific literature on animal health and production. Structured combinations of keywords and Boolean operators were used in both English and Spanish (including terms such as “guinea pig,” “zoonotic disease” “parasites,” “Leptospira,” “Toxoplasma,” “infectious diseases,” “viruses,” “bacteria,” “fungi,” “pathogens”) to identify studies relevant to a One Health framework. The search focused on peer-reviewed articles, prioritizing original research articles involving guinea pigs raised as livestock. Articles not related specifically to livestock guinea pigs or from non-peer-reviewed sources were excluded.

## Guinea pigs as reservoir for zoonotic diseases

As it is detailed in Table 1, following the bibliographic search strategy described above, we included in this review a total number of 29 publications from years 2012 to 2025 about zoonotic diseases associated to livestock guinea pigs. These reports come mainly from Andean countries: Colombia (3 articles; 10.3%), Ecuador (12 articles; 41.4%) and Peru (12 articles; 41.4%) where production of guinea pigs is massive. But there are also reports from African countries like Cameroon (2 articles; 6.9%) where guinea pigs breeding was recently introduced.

**Table 1:** List of publications related with the identification of zoonotic pathogens in guinea pigs raised as livestock included in this review.

Pathogen	Relevance	References
Influenza A	Responsible for seasonal flu outbreaks affecting humans but also poultry	Leyva-Grado et al. (19) and Salas-Rueda et al. (4)
*Staphylococcus aureus* (MRSA), *Streptococcus pneumoniae*, and *Streptococcus pseudopneumoniae*	Responsible for community acquired pneumonia, with some strains specifically associated to livestock	Zambrano-Mila et al. (20), Chaves-Velasquez et al. (45), and Rodriguez-Pazmiño et al. (10)
Zoonotic yeast (*Candida*, *Criptococcus* and others)	Opportunistic respiratory infections in immune depressed individuals	Buela et al. (11)
*Campylobacter jejuni, C. coli, Escherichia coli* and *Salmonella typhimurium*	Enteric bacteria responsible for water andfoodborne gastrointestinal diseases	Vasco et al. (22), Graham et al. (21), Carhuaricra et al. (46), Hurtado et al. (47), and Gamarra et al. (48)
*Protozoa (Giardia, Cryptosporidium, Blastocystis, Entomoeba) and helminths enteric parasites*	Enteric parasites responsible for water andfoodborne gastrointestinal diseases	García et al. (24), Vargas et al. (29), Payne et al. (26), Meutchieye et al. (17), Ríos et al. (27), Gonzalez-Ramírez et al. (49), Gálvez et al. (23), Tacilla et al. (28), and Salas-Rueda et al. (3)
*Toxoplasma gondii*	Foodborne parasite responsible for toxoplasmosis, a systemic disease also causing abortion	Cañón-Franco et al. (30), Roller et al. (31), and Salas-Rueda et al. (4)
*Streptococcus zooepidemicus, Staphylococcus* sp. and *Corynebacteriu*m sp	Pyogenic bacteria associated with cervical lymphadenitis in guinea pig involved in septicemic infections in humans and livestock	Calderón-Ruiz et al. (50), Jara et al. (32), and Ordóñez et al. (33)
*Bartonella rochalimae*, *B. henselae*, and *B. clarridgeiae*	Bacteria responsible for the systemic disease of bartonelosis or cat scratch disease	Rizzo et al. (36)
*Leptospira interrogans*	Responsible for the neglected tropical disease leptospirosis that affects human and domestic animals	Benavides-Benavides et al. (34), Carrión-Montaño et al. (35), and Salas-Rueda et al. (37)

It has been shown that guinea pigs are zoonotic reservoirs of respiratory pathogens of public health concern including Influenza A (19), Methycilin Resistat *Staphylococcus aureus* (MRSA) (20), or antibiotic resistant strains of *Streptococcus pneumoniae* and *S. pseudopneumoniae*, *S. oralis,* and *S. mitis* (Rodriguez-Pazmiño et al., 2025). Moreover, a recent study carried out in Ecuador showed a high prevalence of nasal carriage of zoonotic yeast belonging to eight diferent genera like *Wickerhamomyces, Diutina, Meyerozyma, Candida, Pichia, Rhodotorula, Galactomyces*, and *Cryptococcus* (11).

Guinea pigs are also potential zoonotic reservoirs of enteric bacterial pathogens like *Campylobacter jejuni* or *C. coli*, found at much higher prevalence than in other domestic animals (21, 22). It has also been reported the carriage of zoonotic enteric parasites at high prevalence like *Giardia* and *Cryptosporidium*, and also other like *Blastocystis, Entamoeba, Endolimax, Enteromonas, Retortamonas, Chilomastix, Eimeria, Strongylida and Hymenolepis*, and nematods like *Heligmosomoides polygyrus* (3, 17, 23–29).

Three very recent studies from Colombia, Peru and Ecuador have described for the first time the potential role of guinea pigs as reservoirs for *Toxoplasma gondii* (4, 30, 31). These findings were especially worrisome for three reasons. First, the prevalence of *T. gondii* was very high, with values of 23.3% for the study done in Cuzco (Peru), 27.5% for the study done in Nariño (Colombia), and 16.25% for the study done in Azuay (Ecuador) (4, 30, 31). Second, those prevalence values were based on the detection of DNA from *T. gondii* by PCR confirming the active infection with the parasite. Third, DNA from *T. gondii* was found across multiple tissues, especially in brain, but also in heart and muscle (30, 31). Those findings have strong implication for food safety as guinea pigs are traditionally cooked and consumed including their heads.

*Streptococcus zooepidemicus* is an emerging zoonotic pathogen involved in septicemic infections in humans and livestock, that has also been described in livestock guinea pigs from Peru and Ecuador (32, 33). A highly pathogenic strain of *S. zooepidemicus* carrying important virulence factors was isolated from cervical and mandibular abscesses from guinea pigs following an outbreak of severe lymphadenitis (32).

Finally, two endemic and neglected infectious diseases in the Andean countries have also been linked to guinea pigs farming: bartonelosis and leptospirosis (34–37). One of those studies was carried out in Peru, and a high prevalence of *Bartonella* DNA was found either in guinea pigs blood or fleas, and several species like *B. rochalimae*, *B. henselae* and *B. clarridgeiae* were identified (36). Interestingly, this study was done with guinea pigs raised for self consumption within the household, specifically in the kitchen; those findings underscore the risk for outbreaks of cat scratch disease in humans associated to guinea pigs breeding (36), as it has been recently showed for stray cats in Ecuador (38). Leptospirosis is endemic to tropical settings and cause seasonal outbreaks in humans in Ecuador, Colombia or Peru (38). Moreover, the rodents are considered the mains reservoir of this disease (38). A recent report carried out in Colombia has identified the *Leptospira interrogans* in kidney tissues from guinea pigs at a prevalence of 1.5% (34). However, a recent study carried out by our research group in Ecuador has found a high prevalence and diversity of *Leptospira* pathogenic serovars. Moreover, *Leptospira* DNA was found at high positive rates of 27.7 and 78.7% in urine and fecal samples, respectively, from guinea pigs raised within the household in rural communities from Ecuador, underscoring a potential role of guinea pigs in the transmission dynamics of leptospirosis in the Andean Region.

## Improving livestock guinea pig health from a One Health perspective

Improving livestock guinea pig health requires a comprehensive “One Health” approach that explicitly integrates animal health and human health, but also environmental sustainability. The evidence presented in recent studies and field investigations conducted in the Andean Region demonstrates that the traditional systems in which guinea pigs are raised especially backyard and small-scale family production create biological, environmental, and occupational conditions that facilitate the emergence and transmission of zoonotic diseases. Addressing these challenges demands multisectoral interventions that move beyond animal-level management and incorporate public health, environmental stewardship, and antimicrobial resistance (AMR) mitigation strategies (4, 20, 37).

Biosecurity remains one of the weakest components of guinea pig husbandry in Andean countries. Smallholder systems typically lack controlled access, appropriate waste handling, sanitary barriers, and structured cleaning and disinfection programs (7, 20). These deficiencies directly contribute to the high prevalence of enteric and respiratory pathogens (3, 4, 8–11, 20–22, 30, 37). Improved biosecurity should include controlled entry and exit of people, animals, and equipment; use of personal protective equipment for farmers; compartmentalization of production areas; systematic disinfection protocols; routine sanitary supervision. These actions would significantly reduce pathogen circulation and exposure risk for both animals and farmers (39).

A genuine One Health framework must also address the environmental consequences of guinea pig farming, which have been historically overlooked. In backyard systems, manure, urine, and wastewater are frequently discharged directly onto soil or into nearby water sources, contributing to environmental contamination with zoonotic pathogens and antimicrobial residues. (4, 37). For instance, our recent study demonstrates that guinea pigs shed *Leptospira* through both urine and feces, confirming their role as active environmental reservoirs. Such contamination elevates the risk of infection for humans, and others domestic animals and wildlife (37). To prevent environmental pollution, it is suggested that construction of adequate manure management systems such as biodigesters or composting units; avoidance of direct disposal of waste near water sources; promotion of sustainable land use practices to reduce soil and water contamination; improving ventilation, drainage, and humidity control in farms to limit pathogen survival.

Additionally, the presence of dogs, cats and other domestic animals that interact within farms, frequent in Andean rural communities must be controlled, as they serve as mechanical or biological vectors in the epidemiological cycles of leptospirosis, toxoplasmosis, bartonellosis, and other zoonoses (4, 9, 10, 36, 37).

All the above said, guinea pig farming poses tangible occupational and community health risks. Farmers are routinely exposed to pathogens through direct handling, slaughtering, and cleaning of farms, often without sanitary practices. Families, particularly children and elderly individuals, may also be exposed due to the common practice of keeping guinea pigs inside homes or in adjacent kitchens, as documented in Peru and Ecuador. To mitigate these risks, public health strategies should include farmer training programs, standardized slaughter and meat-handling protocols, community-based education campaigns (8–10, 25, 30). Furthermore, guinea pigs should be included in national programs for surveillance and control of infectious diseases that affect livestock, as it happens with cattle, pigs or poultry. For instance, the current panzootic caused by H5 Influenza that is affecting the Americas has already caused outbreaks not only in wild birds and poultry, but also in many mammal species including dairy cows (40–42). Considering that Influenza A has been already found in guinea pigs in Ecuador, and that small mammal massive production has been associated to explosive H5 influenza outbreaks like for minks in Europe (43), sentinel surveillance of guinea pig farms should be integrated with poultry farms surveillance. All the stakeholders, from producers to authorities, should work together to design and implement policies that promote health within guinea pig production systems within the One Health approach.

AMR is an emerging critical component of the One Health framework, and the Andean guinea pig sector is particularly vulnerable due to unregulated antibiotic use, frequent self-medication, and limited veterinary oversight. It has already been documented the presence of MRSA and resistant *Streptococcus* strains in livestock guinea pigs, reinforcing the urgent need to address antimicrobial practices (8–10, 20). Key AMR-related challenges include widespread access to over-the-counter antimicrobials without prescription, empirical antibiotic use for diarrhea and respiratory diseases, lack of diagnostic confirmation before treatment, absence of withdrawal periods for meat intended for consumption and an overall limited farmer awareness of AMR risks. It is urgent to implement AMR control programs aligned with World Animal Health Organization guidelines, restrict access to certain classes of antibiotics and promote prescription-based use, based on a strengthen laboratory diagnostics in rural regions. It is also crucial to incorporate AMR surveillance in guinea pigs in national monitoring programs for small livestock strengthening the work of the official veterinary service. Moreover, promoting non-antibiotic alternatives, including vaccination, improved nutrition, and enhanced biosafety measures are also strategies to fight AMR threat.

## Concluding remarks

All in all, sustainable improvement of guinea pig production requires coordinated efforts among veterinary services, public health institutions, environmental agencies, universities and communities of breeders and consumers. Technical training, access to veterinary care, incentives for infrastructure improvement, and community participation are essential to strengthen resilience and reduce zoonotic risk (39, 44).

In conclusion, although livestock guinea pigs are reservoirs for zoonotic transmission, these issues could be solved and managed as it is done for other livestock species. Promoting sustainable guinea pig production offers multiple benefits, including enhanced public health, improved animal welfare, and greater food security for communities that rely on this activity. Raising guinea pigs in proper, disease-free conditions bolsters confidence in their role as a safe, high-quality protein source, particularly important for developing countries aiming to export to high-income markets. This holistic approach, which integrates health, sustainability and community development, positions guinea pig production as a strategic driver for improving the quality of life in rural communities in South America and also in Africa where it is an important industry.
